# Cross-Domain Human Activity Recognition Using Low-Resolution Infrared Sensors

**DOI:** 10.3390/s24196388

**Published:** 2024-10-02

**Authors:** Guillermo Diaz, Bo Tan, Iker Sobron, Iñaki Eizmendi, Iratxe Landa, Manuel Velez

**Affiliations:** 1 Department of Communications Engineering, University of the Basque Country, 48013 Bilbao, Spain; inaki.eizmendi@ehu.eus (I.E.); iratxe.landa@ehu.eus (I.L.); manuel.velez@ehu.eus (M.V.); 2 Department of Electrical Engineering, Tampere University, 33100 Tampere, Finland; bo.tan@tuni.fi; 3 Department of Computer Languages and Systems, University of the Basque Country, 48013 Bilbao, Spain; iker.sobron@ehu.eus

**Keywords:** prototypes network, low-resolution infrared, cross-domain, human activity recognition, few-shot learning, recurrent convolutional network, long short-term memory networks

## Abstract

This paper investigates the feasibility of cross-domain recognition for human activities captured using low-resolution 8 × 8 infrared sensors in indoor environments. To achieve this, a novel prototype recurrent convolutional network (PRCN) was evaluated using a few-shot learning strategy, classifying up to eleven activity classes in scenarios where one or two individuals engaged in daily tasks. The model was tested on two independent datasets, with real-world measurements. Initially, three different networks were compared as feature extractors within the prototype network. Following this, a cross-domain evaluation was conducted between the real datasets. The results demonstrated the model’s effectiveness, showing that it performed well regardless of the diversity of samples in the training dataset.

## 1. Introduction

In recent years, the use of machine learning techniques to model and interpret different sensor data for the analysis of human activity has become a prolific field of research, due to its value in various applications such as healthcare, sports, robotics, and security. In particular, in healthcare, long-term activity records are valuable data for identifying early signs of cognitive aging problems; the accurate and timely detection of falling or freezing of gait (FOG) from sensor data could be life-saving techniques for elderly care and emergency response.

Sensor-based human activity recognition (HAR) technologies can be divided into three main groups: (i) based on image/video; (ii) based on inertial sensor data in wearable devices; and (iii) based on wireless radio signals. Compared with wearable-based approaches, noninvasive methods based on video or wireless radio signals require less participation from subjects, and therefore are suitable for dependent individuals. Recently, the wireless-radio-signal-based approach [1] has attracted increasing attention due to its privacy-preserving nature and independence from light conditions. These characteristics make wireless-signal-based sensing particularly suitable for residential environments, which is the main site of future healthcare [2], compared to video-based counterparts.

WiFi-based HAR, with its ubiquity and privacy-respecting nature, has many strengths, including its resilience against environmental factors such as temperature fluctuations and not requiring line of sight. However, the use of wireless signals, generally using channel state information (CSI), is a complex problem [3]. While WiFi has demonstrated significant success, particularly in areas like activity recognition [4] and fall detection [5], our work on low-resolution infrared (LRIR) sensors offers a complementary solution, rather than a competing one. LRIR sensors provide advantages in specific use cases where installation complexity and environmental constraints may be minimal concerns, and they offer unique privacy-preserving benefits that are inherently tied to their low resolution regarding other video-based solutions. Moreover, LRIR sensors have the added advantage of being less susceptible to electromagnetic interference or noise from other wireless signals, which can be a challenge in crowded or signal-dense environments where WiFi-based systems might face performance degradation. In addition, while WiFi-based systems, particularly those using CSI, can be sensitive to environmental changes due to fluctuations in the wireless channel, LRIR sensors offer more stable performance in dynamic environments by directly detecting thermal patterns, although they still require an unobstructed line of sight.

The use of LRIR sensors to recognize human activities has been investigated in previous works. Jeong et al. [6] proposed a probabilistic method with multiple image processing techniques interpolating 8 × 8 data. In this work, the original 8 × 8 thermal pixels formed the heat signature of the human subjects, and they were interpolated to 29 × 29 pixels to apply Gaussian filtering to remove high-frequency noise due to temperature fluctuations. Mashiyama et al. [7] proposed a method for activity recognition using the temperature distribution obtained from an 8 × 8 LRIR sensor. Burns et al. [8] employed two 32 × 31 LRIR sensors to detect daily activities in a kitchen, comparing ground frames with activity frames and using a random forest model to classify. Fan et al. [9] made a comparison between gated recurrent units (GRUs) and long short-term memory (LSTM) models to classify fall detection using an 8 × 8 LRIR sensor. Yin et al. [10] used a noise reduction filter and an LSTM model to automatically extract features from an 8 × 8 LRIR image and build a recognition model. Karayaneva et al. [11] thoroughly investigated noise removal, feature extraction, and neural network models for LRIR data for HAR.

Since high accuracies have been obtained in recognizing human activities using data from the same scenarios, the next step is to achieve similar accuracies using data from different scenarios, days, and conditions, using transferability strategies. In this sense, recent research efforts have been directed towards achieving transferability of results between different environments, often referred to as cross-domain. In this context, the term ‘domain’ refers to the setup, the environment, and the conditions under which the measures were taken. Based on this, cross-domain implies that a model trained with data from a specific condition, e.g., a particular day, room, subject, and/or under particular conditions, is able to correctly classify input data under other conditions.

Although cross-domain HAR is being extensively explored in WiFi- and video-based systems, its application to LRIR sensors remains underexplored. This represents a significant research gap, as the ability to transfer knowledge across domains is essential for the wider adoption of LRIR-based HAR systems. To address this, our paper introduces a novel model aimed at bridging this gap and enhancing the transferability of LRIR-based HAR.

Some deep learning (DL) strategies have been designed for this objective [12], and one of the most prolific is few-shot learning (FSL). FSL allows cross-domain adaptation for a few labeled samples from one dataset using a pretrained model with an extensive dataset. Yin et al. [13] used a semi-supervised cross-domain neural network based on 8 × 8 LRIR images to accurately identify human activities. This network consisted of a convolutional network (CNN) as a feature extractor, followed by two fully connected networks (FC): one two-layer FC for domain discrimination, and another three-layer FC as a label classifier, respectively. This network was a domain adversarial neural network and, in this case, incorporated an FSL strategy in which a small number of the testing samples were labeled for training.

One of the most prominent methods for FSL is prototype networks (PN). These networks are based on learning a prototypical representation for each class, which allows the classification of new samples by comparing them with these prototypes in an embedding space. In the same way, the network employs an embedding function to learn the specific representation of the classes, which is the core of the model. This approach improves the model efficiency by requiring less labeled data and facilitates further generalization to new classes that were not seen during training [14]. The PN most commonly used in the literature uses CNNs as an embedding function to extract features [15,16,17,18,19,20].

A prototype recurrent convolutional network (PRCN) is a type of PN which employs a sequence of convolutional blocks and LSTM cells, combining the CNN and LSTM layers as an embedding function. This CNN-LSTM scheme, also called a long-recurrent convolutional network (LRCN), was used for the first time in [21] as a feature extractor for video-based activity recognition. This showed that an LRCN fed with several frame packets as input greatly improved on the previously employed CNN single-frame baseline method for this task, using its LSTM cell capacity to learn to recognize and synthesize temporal dynamics for tasks involving sequential data, as mentioned previously. Since then, the LRCN has become a popular model in many fields, mainly where CNNs have obtained good results previously and involving nonstationary events [22,23].

PRCNs have been previously used in various works. In [24], the authors employed a CNN-FC-BiLSTM scheme as an embedding function to extract features from X-band SAR images in the MSTAR dataset. In [25], the authors used a one-dimensional convolutional layer and two Bi-GRU units as embedding functions to classify speech imagery data, which were gated recurrent units, an earlier and straightforward model in which LSTM units are an evolved stage. In [26], the authors developed a similar network based on BiLSTM-CNN-FC to classify different types of medical data with temporal sequences.

To address the LRIR cross-domain gap, our work proposes a cross-domain model for LRIR employing a novel PRCN. This PRCN uses a sequence of convolutional, long short-term memory, and fully connected layers as embedding functions to generate the prototypes, as explained in the following sections. In addition, we compared the results with two other PNs: a prototype convolutional network (PCN) and a prototype recurrent network (PRN), which employed convolutional and recurrent layers, respectively, as embedding functions. The results confirmed that the proposed model was robust and presented a transferability capacity specific for HAR data recorded with LRIR sensors. The model achieved an accuracy greater than 90% in the identification of activity and a significantly accurate performance (up to 85%) when the pretrained model was used in a dataset from multiple domains.

The remainder of this paper is organized as follows. Section 2 explains the three prototype networks used in the work. Section 3 describes the two datasets used to test the models and the cross-domain between them. Section 4 covers the evaluation method, including the data selection to train the networks and parameter values. Finally, Section 5 discusses the results and Section 6 presents the conclusions.

## 2. Prototypical Networks

This section explains how a prototype network works from a general point of view. Then, in each subsection, we describe the three different prototypical networks employed in this work, which have different input format data and can extract different features.

**Figure 1:**
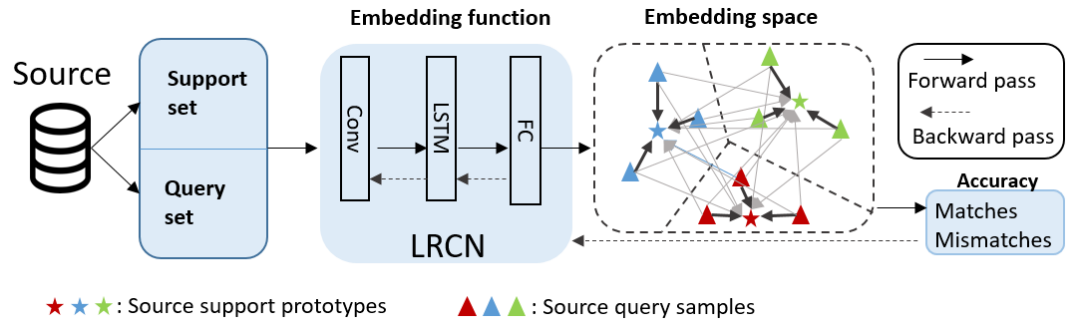
The model uses data from the support set to generate the prototypes and classify the samples from the query set. The accuracy is measured using the number of matches in the last step. Then, the model updates the network weights of the embedding function in a new iteration. The embedding function corresponds with the PRCN model.

In general, a prototype network is based on the idea that it can generate a prototype $c_{k}$ that fits each class *k*. To do so, the network searches for the optimal X-dimensional space in which the samples used as inputs in the training stage are closer to the prototype than the others. The embedding space is searched by updating the embedding function in each iteration. In this sense, each iteration consists of selecting a certain number of samples from each class and dividing them into a support set *S* and a query set *Q*. The samples in *S* generate the prototypes; each prototype is the average of their class samples in the support set. Then, the Euclidean distance between each sample of *Q* and each prototype is measured, obtaining the number of matches and misses. A scheme of this process is shown in Figure 1.

Mathematically, each prototype is the mean vector of the embedded points of a support set $S_{k}$ belonging to a certain class $k\in \left\{1,\ldots ,C\right\}$ such that

(1) $$c_{k}=\frac{1}{|S_{k}|}\sum_{x_{i}\in S_{k}}f\left(x_{i}\right)$$

where $|S_{k}|$ is the cardinality of the support set $S_{k}$ and f(·) is the embedding function. Therefore, assuming a query set of unlabeled samples $Q=\left\{x_{1},x_{2},\ldots ,x_{Q}\right\}$, classification for a given sample $x_{i}$ is carried out by finding the minimum distance to the prototypes, as follows:

(2) $${\hat{y}}_{i}=\arg\min_{k\in \left\{1,\ldots ,C\right\}}\left|\right|x_{i}-c_{k}{\left|\right|}^{2}$$

where ${\hat{y}}_{i}$ is the estimated class for the sample $x_{i}$. Note that any input $x_{i}$ is classified into one of the classes *k*, even though it does not belong to any of them.

The embedded function in a prototype network is a neural network that searches for the optimal embedded space in which the samples of the query set are clustered together with the prototype. The search is based on updating the weights in each iteration. This means that finding the optimal embedding space depends on the ability of the neural network as an embedded function.

### 2.1. Prototypical Convolutional Network

In our work, the PCN model is based on [15]. The embedding function consists of four convolutional blocks. Each convolutional block consists of a 2D convolutional layer, a dropout layer (10%), a batch normalization layer, and a final 2D max-pooling layer. The convolutional layer extracts features from the input image (or from the output of the previous layer in deeper blocks of the network). The dropout layer shuts down specific neurons in each training iteration and is used to avoid overfitting the network to the incoming data and improve generalization capability. The normalization layer adjusts the mean and variance of the data to the same values for each input batch. The final max-pooling layer is used to reduce the dimensionality of each block’s output and reduce the output’s dimensions, while preserving the dominant features. The output of the last convolutional block is vectorized and this vector is used as input to the FC layer, which in our model generates an output of 32 dimensions.

**Figure 2:**
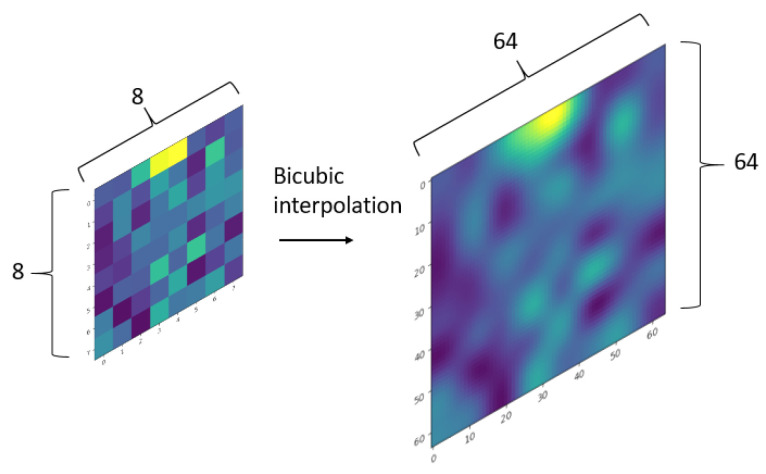
Example of bicubic interpolation from 8 × 8 to 64 × 64 LRIR.

In this model, each 8 × 8 LRIR image was resized to 64 × 64 using bicubic interpolation with a shape 64 × 64, as is shown in Figure 2, and each of them was used as an input. This interpolation was useful to apply more convolutional blocks, as they sequentially reduce the image dimension. The main reason behind the use of bicubic interpolation instead of other interpolation methods (such as bilinear) was that bicubic interpolation tends to generate smoother and more accurate images, as it takes into account a wider area around each pixel compared to the bilinear interpolation. This is especially important when working with low-resolution images such as those from infrared sensors, where details are limited and noise or temperature fluctuations can significantly influence image quality [27,28].

The PCN embedding function is depicted in Figure 4a.

### 2.2. Prototypical Recurrent Network

The PRN employs two consecutive long short-term memory (LSTM) layers as the embedding function. LSTM networks are a type of recurrent neural network (RNN) designed to capture temporal dependencies and sequence information, which makes them particularly well suited for handling sequential data or time series inputs [29]. Each LSTM layer in the PRN is equipped with a set of gates (input, forget, and output gates) that regulate the flow of information, allowing the network to maintain long-term dependencies and mitigate the vanishing gradient problem commonly encountered in traditional RNNs. In the PRN, the input data are first fed into the initial LSTM layer. This layer processes the input through internal mechanisms, generating hidden states that encapsulate the current input and the accumulated knowledge from previous time steps. The output of the first LSTM layer is then passed on to the second LSTM layer, which further refines and processes the information. This stacked LSTM configuration enhances the model’s ability to learn complex patterns and dependencies within the data. The final output of the second LSTM layer is a 32-dimension vector representation that summarizes the entire input sequence. This vector is used as input for the FC layer, which generates the output with 32 dimensions.

**Figure 3:**
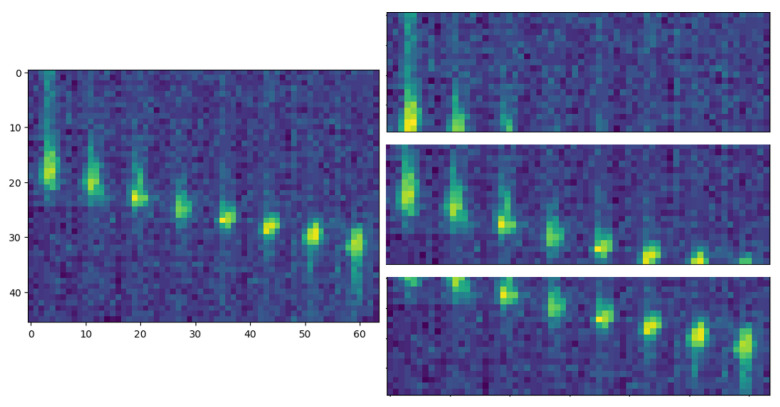
Example of a vectorized spatiotemporal 2D array. The original image was divided into three images with a shape of 20 frames × 64 pixels. Each row corresponds to one flattened 8 × 8 LRIR.

**Figure 4:**
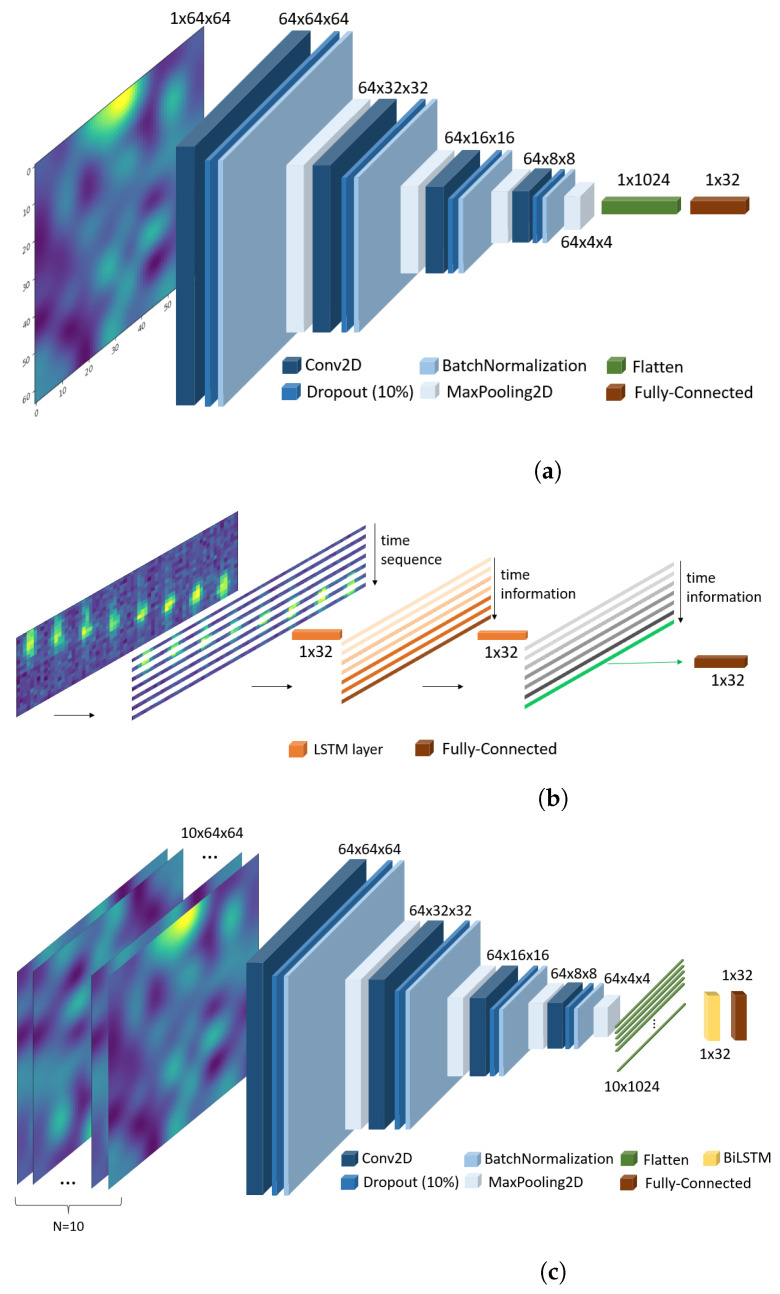
Embedding functions for the three prototypical networks used in this work: (**a**) PCN (prototypical convolutional network), (**b**) PRN (prototypical recurrent network), and (**c**) PRCN (prototypical recurrent convolutional network).

Regarding the PRN input, the LRIR images were converted to spatiotemporal matrices over 20 frames and 64 pixels with a shape 20×64, in which each 8 × 8 LRIR image was flattened and 20 of these consecutive vectors were grouped to form spatiotemporal matrices. The number of 20 consecutive samples was obtained experimentally. To increase the number of samples, consecutive spatiotemporal samples overlap half of the previous sample, as shown in Figure 3. In this way, each input contains sequential or temporal information about each activity, in line with the mentioned capabilities of the LSTM cells. The PRN embedding function is depicted in Figure 4b.

### 2.3. Prototypical Recurrent Convolutional Network

A PRCN combines the power of a CNN to extract static characteristics and the ability of LSTM cells for temporal sequences, to improve the capabilities of its embedded function. Specifically, our PRCN consisted of four blocks of convolutional layers followed by two LSTM cells and a final fully connected layer. Each convolutional block consisted of a 2D convolutional layer, a dropout (0.1) layer, a normalization layer, and a final MaxPooling layer for dimensionality reduction. The LSTM block consisted of two consecutive LSTM cells of 32 dimensions. Finally, the FC layer generated the output of the 32 dimensions. The PRCN embedding function is depicted in Figure 4c.

Each input in the network was a 10 × 64 × 64 matrix, formed by ten consecutive IR 8 × 8 images of the same class, each of them interpolated to 64 × 64 dimensions as in Figure 2. In the same way as for the PRN, the number of 10 consecutive samples was obtained experimentally.

#### How Did It Perform?

When the 10 × 64 × 64 matrix enters the network, each of the ten consecutive images passes through the convolutional part independently and finally are flattened into a vector with a shape of 1 × 1024. The convolutional blocks highlight the most relevant features, such as edges and heat patterns. In each block, the convolutional layer identifies key spatial patterns, while the max pooling reduces the resolution, keeping only the most dominant features. Once the ten images have been flattened, the ten flattened vectors are grouped to form a sequential matrix of 10×1024 following the order of entry. This matrix contains information about static features from the respective images and forms a temporal sequence.

The two LSTM layers work together to process temporal image sequences. The first LSTM layer receives the sequence matrix of size 10×1024 and captures basic temporal patterns. It adjusts the hidden state in each step through its gating mechanisms (forgetting, input, and output), remembering relevant information and discarding unnecessary information. Ultimately, this layer generates an intermediate representation of the hidden states for each sequence step. The second layer of the LSTM takes the hidden states generated by the first layer and processes them again to further refine temporal dependencies and extract more complex patterns. While the first layer captures short-term relationships, the second layer delves into long-term patterns, generating a final hidden state that encapsulates the key information of the entire sequence. A fully-connected layer then performs activity classification using this final hidden state. This combination of layers allows for better extraction of temporal features, achieving greater accuracy in identifying complex sequences.

Because the recording environment is static in both datasets, the temporal features extracted by the LSTM cells contain more information about temporal sequences strongly related to human activities than can be obtained using convolutional networks alone, improving the model’s cross-domain capability.

## 3. Data Description

In order to prove the capacity of the PCN, PRN, and PRCN using LRIR sensors, two different datasets previously mentioned in the literature [11,30,31] were used: Coventry and InfraADL. Both datasets used the same sensor, the Panasonic^®^ Grid-EYE sensor (AMG8833), with a rate of 10 frames per second (FPS), and collected data in indoor environments with one or two people doing the same or similar activities.

### 3.1. InfraADL

**Figure 5:**
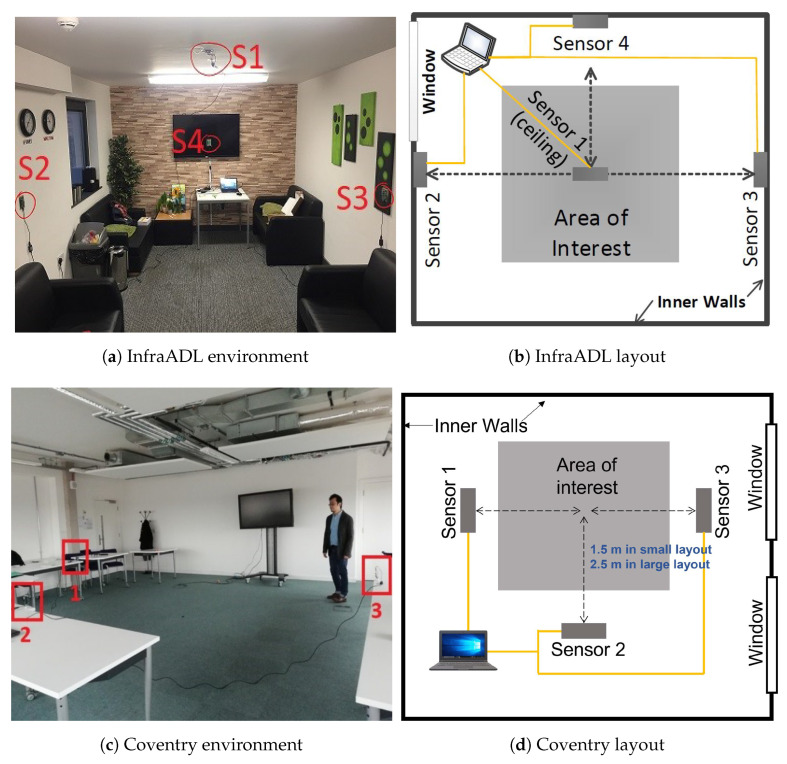
Layouts and environments of two experiment venues.

This dataset was collected at the Bristol Robotic Lab (BRL) at the University of West England. It collected data from four sensors: three on different walls and the last on the ceiling of the room. The layout of the sensors and the experiment environment of the InfraADL dataset are shown in Figure 5a,b.

This dataset contains data for one, two, and three people performing activities simultaneously in the room. The combination of people and activities generates 21 classes to classify. However, our work only considered the data for one and two people and the activities that both datasets have in common to compare the results between them.

In InfraADL, each measurement was repeated three times, with a total of nine people engaged in the activities. Consequently, the measures involving only one person were performed by each individual three times, while the measures involving two were repeated three times for each combination of the nine people.

### 3.2. Coventry

The Coventry dataset was collected at the Faculty of Engineering, Environment and Computing at Coventry University. It collected data from three sensors located in three different tables in a room. The layout of the sensors and the experiment environment of the Coventry dataset are shown in Figure 5c,d.

In this case, there are a total of three people performing the activities, and it contains two sizes for the area of interest: in the small layout, sensors were equally placed 1.5 m away from the space assigned for the subject activity, while in the large layout, the distance was 2.5 m. Only one person performed the activities in the small layout, but in the large layout, measurements were taken for one or two people. For our work, we did not differentiate between room sizes; the same activity corresponded to the same class.

### 3.3. Common Classes

To prove the cross-domain capability between the InfraADL and Coventry datasets, both had to contain the same or similar classes. Table 1 shows the correspondence between both the dataset classes. Some of the classes were simplified for the sake of comparison. For example, some classes relating to “walking” in different directions were unified. For more details, the number of samples for each class in both datasets can be consulted in Table 2.

## 4. Evaluation

In this work, evaluation was carried out in two ways. First, a performance comparison of the three prototypical network architectures described in Section 2 was performed, training and testing the models on the two datasets, with 75% of the data to train the model and 25% to test, using cross-validation with five iterations, and with the same number of samples per class in training to prevent overfitting.

Second, the cross-domain performance of the proposed PRCN solution was tested. For this purpose, we separated the two datasets into source and target datasets. The source dataset was the one on which the model was trained, and the target dataset was the one on which the model was validated. Both InfraADL and Coventry datasets were used as sources and targets. Since our model follows an FSL strategy, the target dataset had to have a small number of labeled samples, L=[8,16,32,64,128]. The comparison was made between the model trained directly on the target dataset using *L* samples and the model trained on the source dataset and subsequently retrained on the target dataset using the *L* samples. In this work, samples were randomly selected from the entire dataset.

In addition, fine-tuning was used for the retraining step. Fine-tuning is a technique that consists of freezing the weights of the deep layers of a model and retraining only the shallower layers, thus avoiding the overfitting that can be generated when retraining with few samples and maintaining the learning of the deep layers obtained in the training with an extensive dataset [32]. Following our previous work [4], the weights of the convolutional blocks were frozen, while the weights of the LSTM cells and the FC layer were retrained. With this in mind, we tested whether the model trained on the large dataset was more efficient than the model trained directly on the target dataset using *L* samples.

Table 3 shows the training parameters for the models. Following [14,15], the number of samples in both support and query sets was four, while the other parameters were obtained experimentally.

## 5. Results

This section shows the results of the prototype networks described in Section 2, the PCN, PRN, and PRCN models, on the datasets described in Section 3, InfraADL, and Coventry. First, each model was trained and tested on the same dataset. All sensors were tested individually and in combination with the others. Then, a second step of cross-domain recognition between datasets was evaluated using the PRCN model. The cross-domain recognition was evaluated for the data from Sensor 2 in both datasets according to Figure 5, to simplify the results. The results are given in terms of accuracy and F1 score averaged by the sensor combinations and the five cross-validation iterations. In addition, several confusion matrices are shown to provide more details of the classification performance of the prototype-based models.

### 5.1. Prototype Network Comparison

**Figure 6:**
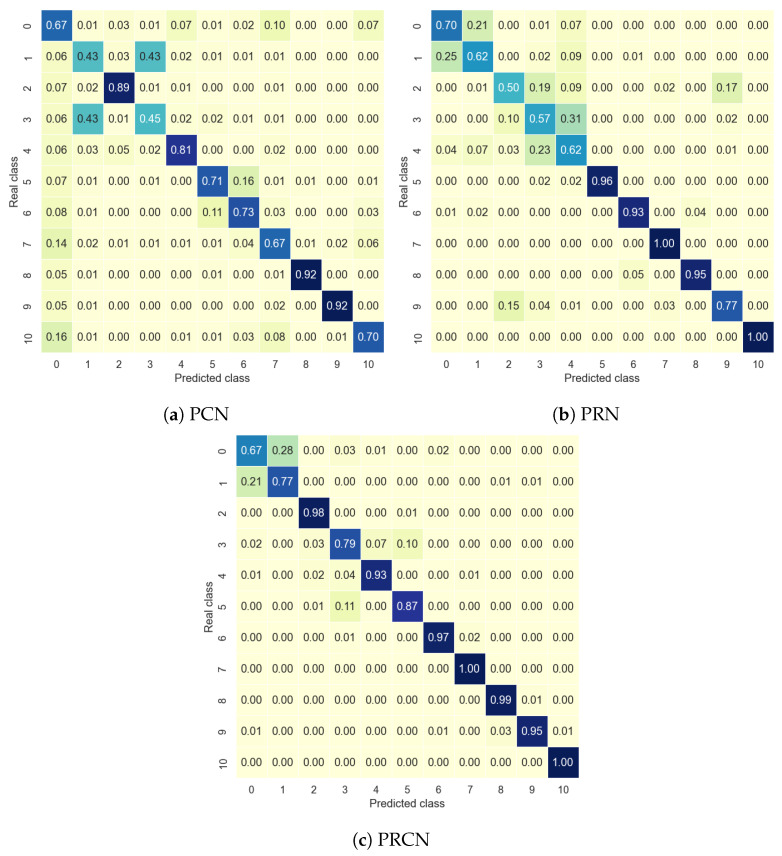
PCN, PRN, and PRCN confusion matrices for InfraADL Sensor 2 data.

In this subsection, the results for the PCN, PRN, and PRCN models on the InfraADL and Coventry datasets are presented in Table 4 and Table 5, respectively. In addition, Figure 6 shows examples of the confusion matrices obtained by the models for the data from Sensor 2 in the InfraADL dataset.

According to Table 4 and Table 5, the PRCN model substantially improved on the accuracy of the PCN and PRN models in both cases, with accuracies greater than 90% in most cases. The lowest values corresponded to the PCN model, so it was demonstrated that using LSTM layers can be a better option for temporal activities than a CNN when using low-resolution images for temporal sequences. In addition, the results in these two tables show that extra sensors gave some performance improvements, but these were not significant. In this case, a single sensor performed similarly for different locations with low position dependence.

Figure 6 shows the accuracy for each class and for each prototypical network. For the PCN and PRN, a relation between the number of people in the scene and the accuracy can be seen, taking into account that for the classes from 0 to 5 there was one person performing the activity, and from 6 until 10 there were two people. Both models seemed to work better for two people than for one person, and a similar but lesser tendency appeared for the PRCN model.

With respect to the number of sensors, there was a slight improvement in accuracy when using more than one sensor.

### 5.2. PRCN Cross-Domain

This subsection presents the results of the PRCN’s capacity for cross-domain recognition. In this sense, the model’s accuracy was tested on the target dataset in two ways: having previously been trained with the source dataset (cross-domain) or without a pretraining step (without cross-domain). Furthermore, as outlined in Section 4, cross-domain fine-tuning of the outer layers was implemented, with the results presented according to the number of samples (*L*) used for retraining.

Table 6 and Table 7 show the accuracy of the Coventry and InfraADL datasets, respectively. In both cases, the pretrained model offered better accuracy than the non-pretrained: following Table 6, for the Coventry dataset, the best improvement was using 8 samples, going from an accuracy of 0.25 to 0.65, and for 128 samples, the improvement was from 0.66 to 0.85. In the same way, following Table 7, for the InfraADL dataset, the best improvement was using 16 samples, passing from an accuracy of 0.33 to 0.61, and for 128 samples, the improvement was from 0.67 to 0.83. Moreover, considering the accuracies for the pretrained models by the number of samples, in the Coventry dataset, an improvement of about 20% could be observed, from 0.65 for 8 samples to 0.85 for 128 samples, while in the InfraADL, the improvement was more significant, about 40%, from 0.45 for 8 samples to 0.83 for 128 samples. The conclusion is that, in general, the authors did not detect a significant effect of using one or the other dataset as the source or target.

**Figure 7:**
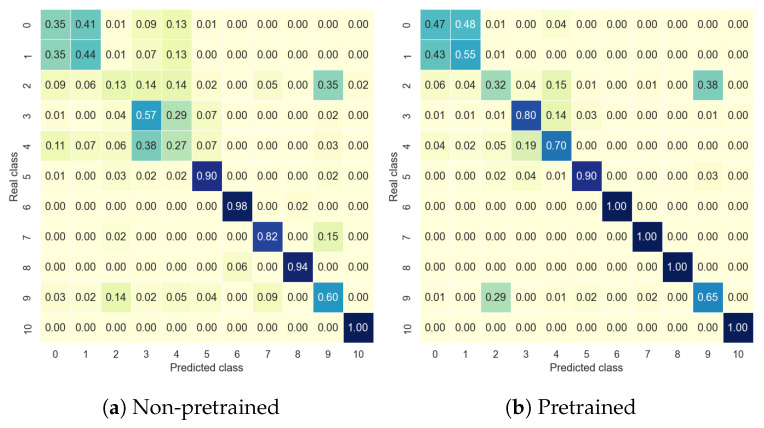
PRCN confusion matrices for Coventry Sensor 2 data with *L* = 64 samples per class, using (**a**) a non-pretrained model, (**b**) a pretrained model with InfraADL sensor 2 data.

Figure 7 offers a detailed class comparison between the pretrained and non-pretrained models for *L* = 64 samples. As can be seen, the accuracies follow the same distribution in both cases; two people’s activities were better recognized than activities performed by one person, and in all cases, the pretrained model improved on the non-pretrained model’s accuracy.

**Figure 8:**
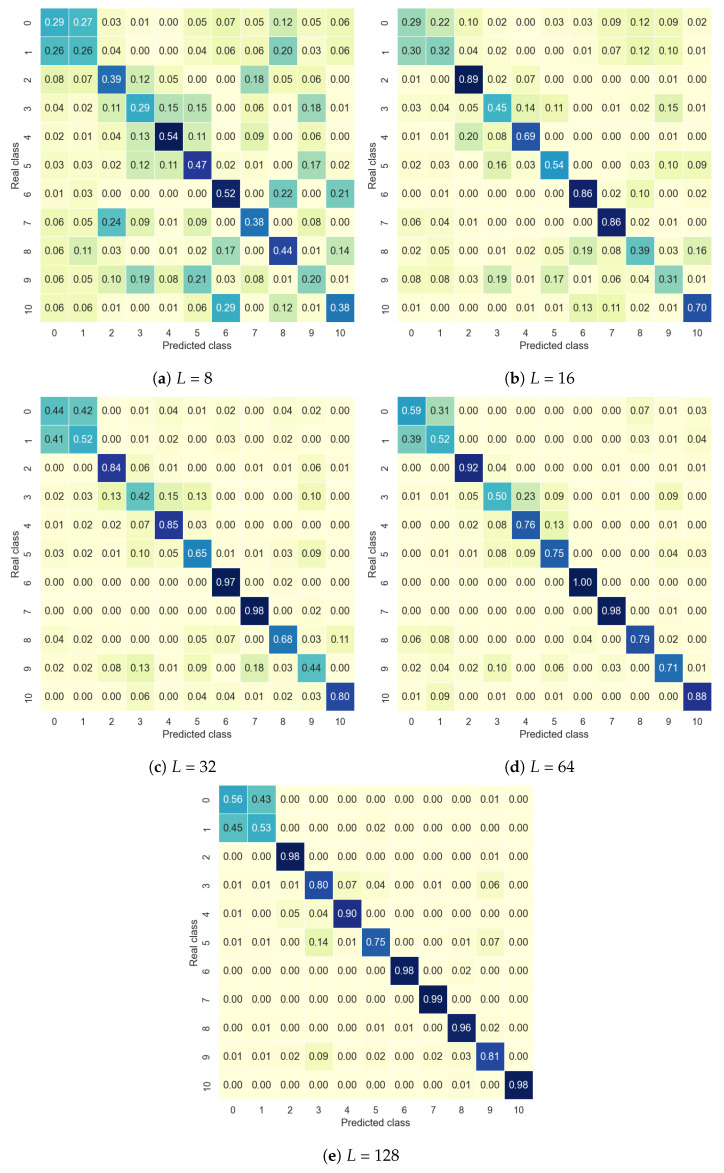
PRCN confusion matrices for InfraADL Sensor 2 data according to *L* (samples per class), using a pretrained model with Coventry Sensor 2 data. These results correspond to Table 7.

A comparison of the number of samples is presented in Figure 8. In these confusion matrices, it can be seen that, in samples *L* = 16, the diagonal for the true positives is clear, although there are notable differences for some classes. For example, classes number 2, 6, and 7, corresponding to the activities stand still, sitting, and standing, respectively, had more than 85% accuracy for *L* = 16. They were the only three activities in which there were no movements.

Following the class analysis, it is worth noting that, due to the activity similarity, there was a general misclassification between class 0 and 1, corresponding to the sitting up and sitting down activities. This probably occurred because of the similar movements involved in these activities. Both classes represent transitions between standing and sitting positions, involving similar movement patterns and thermal changes being captured by the infrared sensors, which made the spatial features extracted by the CNN similar. In addition, both involve fast movements with a short temporal pattern, so the LSTMs could have captured similar activation patterns for both transitions, since both the duration of the action and the gradual changes in the images were almost identical. Since the PRCN generates embedded representations for each class, classes 0 and 1 were likely located very close together in the feature space, due to the high similarity of the inputs. When these two classes are combined, they show an accuracy rate greater than 90% for *L* > 16. According to Figure 7, classes 2 (standing) and 9 (standing and moving), were confused by the network for Sensor 2 of the Coventry dataset. In this case, if the person moving makes small movements, due to the sensor’s low resolution, it may be challenging to differentiate between them. In addition, the sensor’s relative position to the person’s displacement may also make the thermal marks of the two individuals difficult to distinguish. Some similarities occurred for classes 3 and 4, corresponding to one person walking in different directions; even class 5, corresponding to two people walking, could be part of the same group, according to Figure 7 and Figure 8.

Another essential aspect is the impact of the bicubic interpolation used to scale the images from 8 × 8 to 64 × 64. Although this process improved the ability to apply deep convolutions, it can also smooth out specific details, causing features discriminating between similar activities to be lost. This could be especially problematic in activities where the changes are subtle, such as standing and sitting.

It is important to recognize that the two datasets being compared are not fully homogeneous, as they include activities that are not exactly equivalent. As shown in the correspondence of activities in Table 1, the degree of similarity between the activities in the two datasets varies. Although both used the same measurement system, the data were collected by different teams, in different locations, and using slightly different methodologies. These variations in data collection affected the model’s ability to transfer learning from one domain to another, as the conditions under which the activities were recorded were not identical, introducing noise and discrepancies into the patterns the model is trying to learn.

Furthermore, the spatial placement of the sensors is critical, especially for activities involving directional movements, such as moving left to right or forward and backward. In these cases, if the sensors were positioned differently across the datasets, the model may not capture movement patterns in the same way. Unfortunately, there is no direct correspondence between sensor positions in the two datasets, which exacerbated this discrepancy. In this sense, the results suggest that a less detailed grouping of classes—merging those with very similar patterns—could improve the overall accuracy of transfer learning. By reducing the granularity of classification, the model would face a simpler task and could generalize better across domains, mitigating the impact of differences in data collection methods and sensor placement.

## 6. Conclusions

This paper dealt with two LRIR-based HAR datasets to evaluate cross-domain recognition via few-shot learning and proposed a novel prototypical recurrent convolutional network (PRCN).

A first evaluation compared the feature extraction capabilities in the prototype network using three models: a convolutional network (CNN) based on the literature, a recurrent network (LSTM) to take advantage of the time sequence of the data, and a mixture of both using a variant long-recurrent convolutional network (LRCN), which was the model proposed in this work. The results confirmed a relevant improvement for our model and showed a high accuracy, higher than 90% in most classes, verifying that the proposed PRCN is a robust model for HAR recorded with LRIR sensors.

Secondly, a cross-domain evaluation was performed between the two datasets to test the proposed PRCN. Eleven similar activities were evaluated by one or two people in two different scenarios. In general, the results showed a greater capacity for transferability, mainly for activities performed by two people, as well as for those activities that were more static, such as standing still or sitting. In addition, the accuracy could be improved if the transferability was between sensors with the same relative position in the room.

In conclusion, the presented results are promising for the transferability of LRIR-based HAR models between different scenarios. Determining the diversity required for dataset measures to make the model work as well as possible is a key task in developing this field. As future work, it would be valuable to explore how the more advanced development of WiFi-based HAR systems can be combined with and complement LRIR-based approaches, potentially creating hybrid systems that leverage both technologies’ strengths.

## Figures and Tables

**Table 1:** Correspondence of InfraADL and Coventry classes.

Class	InfraADL	Coventry	People
0	Stand to Sit	Sit Down	1
1	Sit to Stand	Stand Up	1
2	Stand Still	Stand Still	1
3	Left to Right Move	Left & Right Move	1
3	Right to Left Move	Left & Right Move	1
4	Move Away	For-Backward Move	1
4	Move Toward	For-Backward Move	1
5	Walking in Opposition	Both Walking	2
5	Walking in Accordance	Both Walking	2
6	Both Sitting	Both Sitting	2
7	Both Standing	Both Standing	2
8	Sitting & Walking in Front	Sitting & Moving	2
8	Sitting & Walking Behind	Sitting & Moving	2
9	Standing & Walking in Front	Standing & Moving	2
9	Standing & Walking Behind	Standing & Moving	2
10	Sitting & Standing	Sitting & Standing	2

**Table 2:** Class distribution by number of 8 × 8 LRIR samples.

Class	InfraADL	Coventry
0	10,684	13,437
1	3040	19,496
2	4572	16,824
3	3244	19,943
4	10,955	12,846
5	8228	4653
6	4628	10,686
7	10,492	8778
8	4352	5131
9	3864	4788
10	9064	5802
Total	73,123	122,384

**Table 3:** Training parameters.

Params	Value
Input shape (PCN, PRN, PRCN)	64 × 64, 20 × 64, 10 × 64 × 64
Learning rate	0.001
Classes number	11
Support set size	4
Query set size	4
Epochs	20
Epoch size	100

**Table 4:** Results for InfraADL dataset.

	Metric	PCN	PRN	PRCN
One sensor	Acc	0.73 ± 0.03	0.83 ± 0.03	0.90 ± 0.01
	F1	0.69 ± 0.03	0.78 ± 0.03	0.89 ± 0.01
Two sensors	Acc	0.76 ± 0.03	0.82 ± 0.05	0.91 ± 0.01
	F1	0.73 ± 0.03	0.78 ± 0.05	0.91 ± 0.01
Three sensors	Acc	0.75 ± 0.02	0.85 ± 0.04	0.93 ± 0.01
	F1	0.71 ± 0.02	0.81 ± 0.04	0.92 ± 0.01
Four sensors	Acc	0.77 ± 0.04	0.85 ± 0.03	0.93 ± 0.01
	F1	0.73 ± 0.03	0.80 ± 0.04	0.93 ± 0.01

**Table 5:** Results for Coventry dataset.

	Metric	PCN	PRN	PRCN
One sensor	Acc	0.70 ± 0.03	0.85 ± 0.03	0.92 ± 0.01
	F1	0.66 ± 0.02	0.79 ± 0.04	0.90 ± 0.01
Two sensors	Acc	0.72 ± 0.03	0.85 ± 0.05	0.92 ± 0.01
	F1	0.70 ± 0.03	0.81 ± 0.03	0.91 ± 0.01
Three sensors	Acc	0.72 ± 0.02	0.85 ± 0.03	0.93 ± 0.01
	F1	0.69 ± 0.02	0.82 ± 0.02	0.92 ± 0.01

**Table 6:** PRCN accuracy values for the Coventry Sensor 2 data according to *L* (samples per class). The pretrained model was previously trained with the InfraADL Sensor 2 data.

*L*	Accuracy	
	Non Pretrained	Pretrained
8	0.23 ± 0.03	0.65 ± 0.05
16	0.33 ± 0.05	0.66 ± 0.05
32	0.50 ± 0.06	0.73 ± 0.06
64	0.63 ± 0.05	0.78 ± 0.05
128	0.66 ± 0.04	0.85 ± 0.03

**Table 7:** PRCN accuracy values for the InfraADL Sensor 2 data according to *L* (samples per class). The pretrained model was previously trained with the Coventry Sensor 2 data.

*L*	Accuracy	
	Non Pretrained	Pretrained
8	0.34 ± 0.11	0.45 ± 0.03
16	0.33 ± 0.06	0.61 ± 0.04
32	0.42 ± 0.07	0.69 ± 0.03
64	0.51 ± 0.06	0.75 ± 0.04
128	0.67 ± 0.09	0.83 ± 0.04

## Data Availability

The Coventry dataset is public and open access: https://ieee-dataport.org/documents/infrared-human-activity-recognition-dataset-coventry-2018, accessed on 30 September 2024. The InfraADL dataset is available under request.
